# Supplementary material for: Heat Shock Factor 1-dependent extracellular matrix remodeling mediates the transition from chronic intestinal inflammation to colon cancer
Source: Nat Commun. 2020 Dec 7;11:6245. doi: 10.1038/s41467-020-20054-x (PMC7721883; doi:10.1038/s41467-020-20054-x)
Supplement: Supplementary file 1 — Supplementary Information [file 41467_2020_20054_MOESM1_ESM.pdf]

Supplementary Information

Title: Heat Shock Factor 1-dependent extracellular matrix remodeling mediates the transition from chronic intestinal inflammation to colon cancer

Oshrat Levi-Galibov[1], Hagar Lavon[1], Rina Wassermann-Dozorets[1], Meirav Pevsner-Fischer[1], Shimrit Mayer[1], Esther Wershof[2], Yaniv Stein[1], Lauren Brown[3], Wenhan Zhang[3], Gil Friedman[1], Reinat Nevo[1], Ofra Golani[4], Lior Katz[5,6], Rona Yaeger[7], Ido Laish[5,8], John A. Porco, Jr.[3], Erik Sahai[2], Dror S. Shouval[8,9], David Kelsen[7] and Ruth Scherz-Shouval[1*]

[1]Department of Biomolecular Sciences, The Weizmann Institute of Science, Rehovot, Israel, [2]The Francis Crick Institute, London, UK, [3]Department of Chemistry and Center for Molecular Discovery (BU-CMD), Boston University, Boston, Massachusetts, USA, [4]Department of Life Sciences Core Facilities, The Weizmann Institute of Science, Rehovot, Israel, [5]Gastroenterology Institute, Sheba Medical Center, Tel Hashomer, Ramat Gan, Israel, [6]Department of Gastroenterology and Hepatology, Hadassah Medical Center, Jerusalem, Israel, [7]Gastrointestinal Oncology Service, Memorial Sloan Kettering Cancer Center, and Weil Cornell Medical College, New York, NY, US, [8]Sackler Faculty of Medicine, Tel-Aviv University, Tel Aviv, Israel, [9]Pediatric Gastroenterology Unit, Edmond and Lily Safra Children's Hospital, Sheba Medical Center, Ramat Gan, Israel.

[*]Correspondence should be addressed to R.S.S (Email: ruth.shouval@weizmann.ac.il)

The Supplementary Information file contains 8 Supplementary Figures and 2 Supplementary Tables.

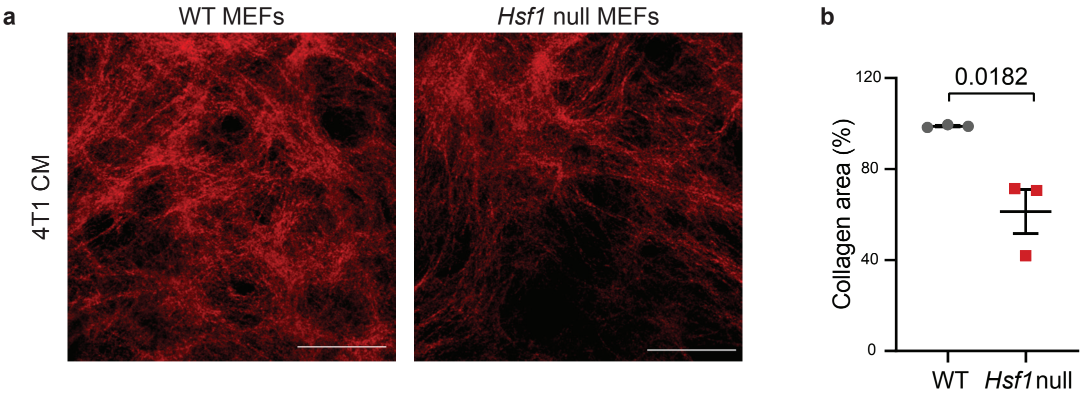

Supplementary Figure 1: *Hsf1* null fibroblasts exhibit impaired ECM secretion. WT or *Hsf1* null primary MEFs were induced to secrete ECM by 12 days incubation with conditioned media from 4T1 breast cancer cells **(a)** supplemented by growth factors and insulin. Representative images are shown in **(a)**. The average area of collagen covered is quantified in **(b)** for 3 technical replicates. The experiment was repeated twice, each with MEFs from different mice, representative results are shown as mean ± SEM. Scale bar – 50 µm. Analyzed by student's t-test (two-sided).

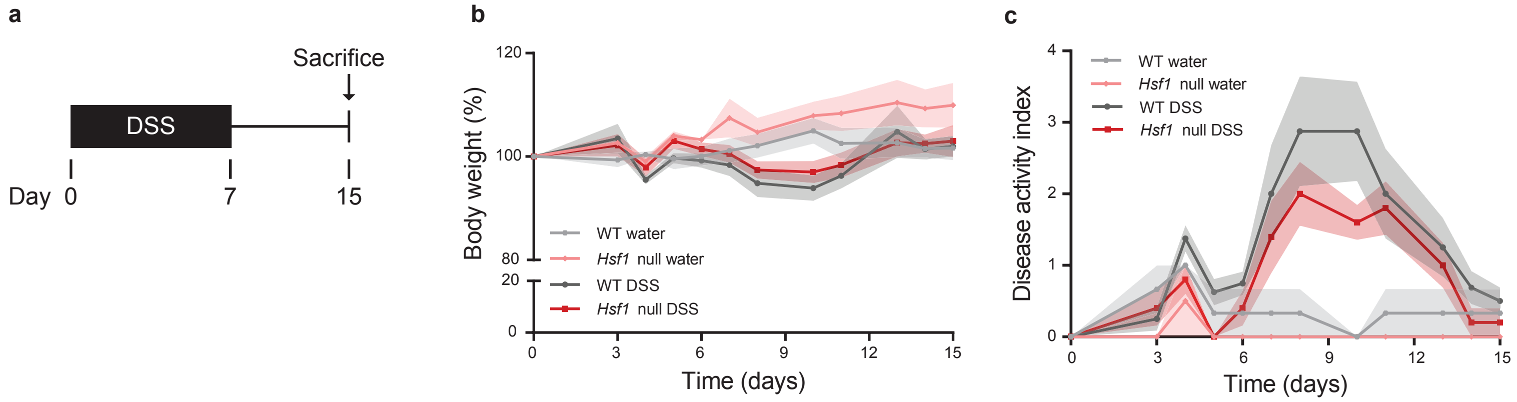

Supplementary Figure 2: *HSF1* status does not affect the response of mice to an acute DSS treatment. (a) Schematic representation of the experimental protocol. WT and *Hsf1* null mice were treated with 1.5% DSS or water for 7 days. **(b-c)** Changes in the body weight of the mice **(b)** and their Disease activity index **(c)** were monitored. Results are presented as mean ± SEM, analyzed by two-way ANOVA, utilizing Tukey's test to account for multiple comparisons for multiple comparisons. n=3 WT mice and n=2 *Hsf1* null mice in water treated groups; n=8 WT and n=5 *Hsf1* null mice in DSS treated groups.

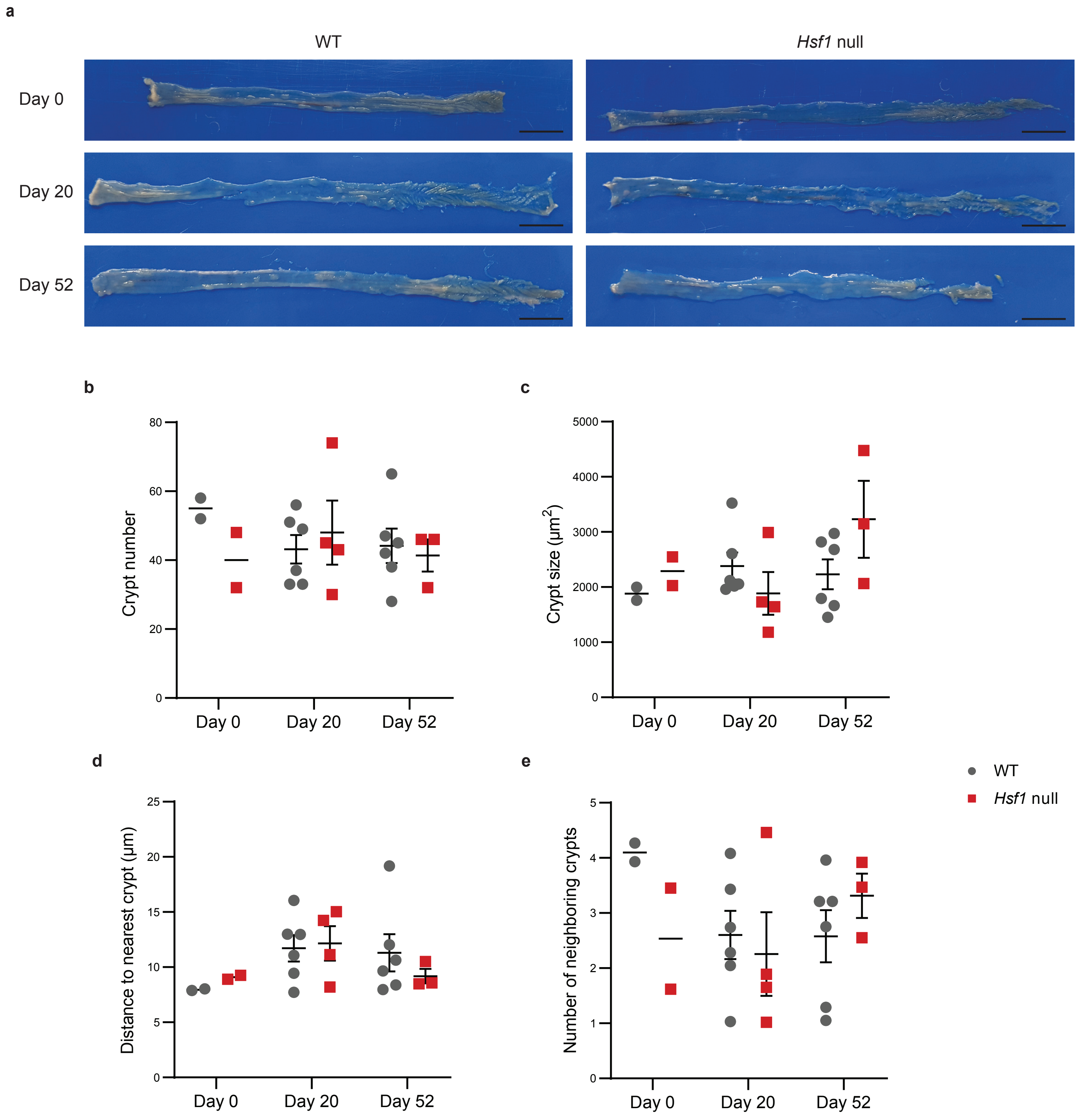

Supplementary Figure 3: Naïve WT and *Hsf1* null mice share similar colon morphologies and crypt structures. WT (left) and *Hsf1* null mice (right) were sacrificed at day 0 or underwent sham treatment (Saline injection, normal drinking water and colonoscopies at day 20 for 20-day control mice, or days 39 and 52 for day 52 control mice). N=6 WT (day 20 and day 52) and n= 4 or 3 *Hsf1* null (day 20 or 52 respectively) sham treated mice; n=2 day 0 control mice for each genotype. **(a)** Representative images of WT (left) and *Hsf1* null (right) colons of naïve mice at time zero (top panels) or at day 20 and 52 of sham treatment (lower panels) are shown. Scale bar - 1 cm. **(b-e)** Quantification of crypt number **(b)**, crypt size **(c)**, distance to the nearest crypt (within 40 µm) **(d)**; and number of neighboring crypts (within 20 µm) **(e)** in mouse colons following 15 and 20 days of AOM-DSS treatment based on SHG images of mice at day 0 or after sham treatment. Results are presented as mean ± SEM, analyzed by two-way ANOVA, and found to be non-significant.

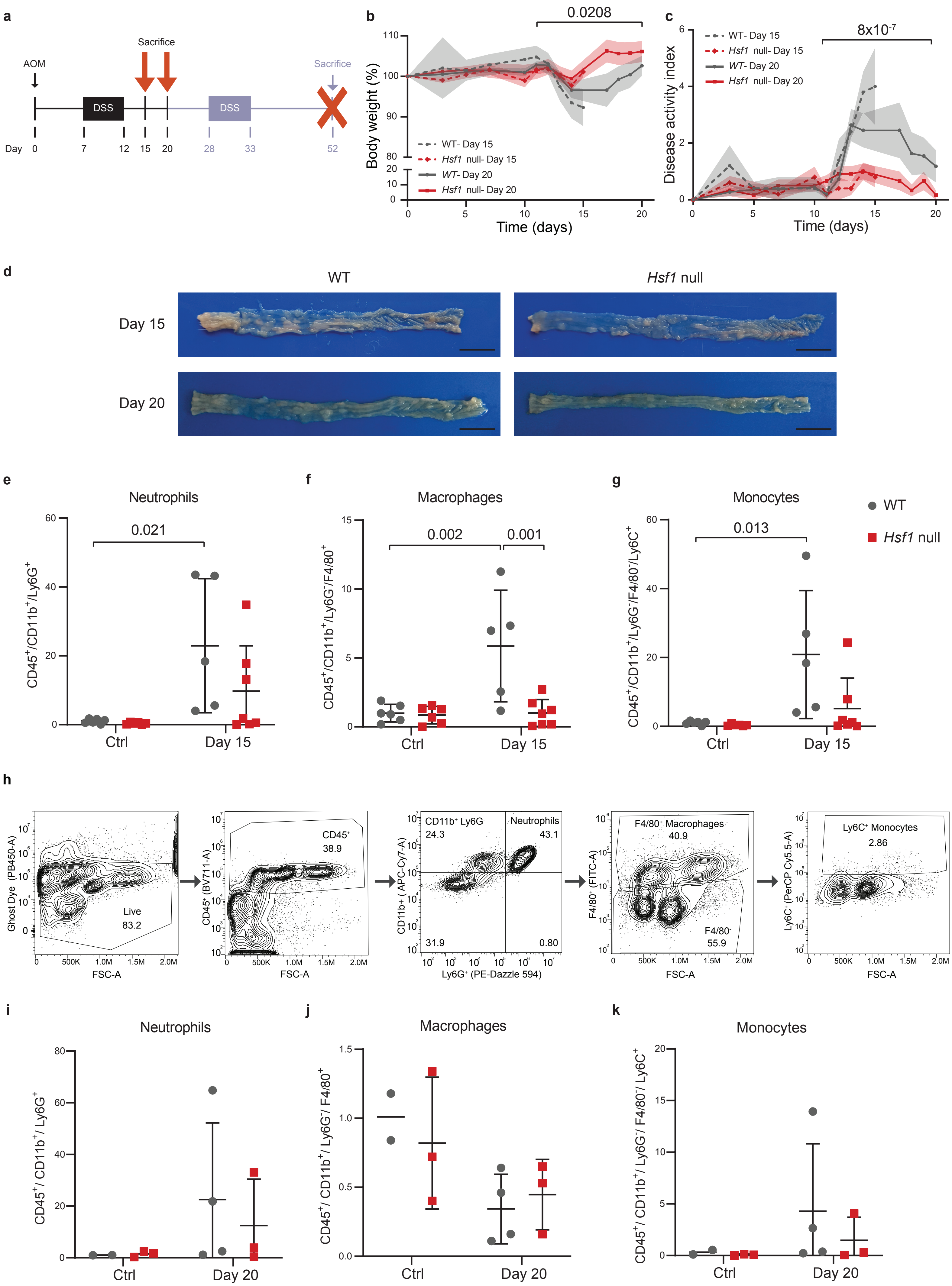

a
AOM
Sacrifice
Sacrifice
DSS
DSS
Day 0 7 12 15 20 28 33 52
b
0.0208
Body weight (%)
Time (days)
WT- Day 15
Hsf1 null- Day 15
WT- Day 20
Hsf1 null- Day 20
c
8x10-7
Disease activity index
Time (days)
d
WT
Hsf1 null
Day 15
Day 20
e
Neutrophils
0.021
CD45+/CD11b+/Ly6G+
Ctrl
Day 15
f
Macrophages
0.002
0.001
CD45+/CD11b+/Ly6G-/F4/80+
g
Monocytes
0.013
CD45+/CD11b+/Ly6G-/F4/80-/Ly6C+
WT
Hsf1 null
h
Ghost Dye (PB450-A)
Live
83.2
FSC-A
CD45+ (BV711-A)
CD45+
38.9
CD11b+ Ly6G-
24.3
Neutrophils
43.1
31.9
0.80
CD11b+ (APC-Cy7-A)
Ly6G+ (PE-Dazzle 594)
F4/80+ (FITC-A)
F4/80+ Macrophages
40.9
F4/80-
55.9
Ly6C+ (PerCP Cy5.5-A)
Ly6C+ Monocytes
2.86
i
Neutrophils
CD45+/ CD11b+/ Ly6G+
Ctrl
Day 20
j
Macrophages
CD45+/ CD11b+/ Ly6G-/ F4/80+
k
Monocytes
CD45+/ CD11b+/ Ly6G-/ F4/80-/ Ly6C+

Supplementary Figure 4: Neither WT nor *Hsf1* null mice exhibit macroscopic tumors at Day 15 or 20 of the AOM-DSS protocol. (a) Schematic representation of the experimental protocol. WT and *Hsf1* null mice (n=5 mice for both genotypes at day 15, n=11 WT mice at day 20 and n=6 *Hsf1* null mice at day 20) were injected intraperitoneally with AOM (10 mg/kg), followed by 5 days of 1.5% DSS in the drinking water, and sacrificed 3 or 8 days later (day 15 and 20, respectively). **(b-c)** Changes in the body weight of the mice **(b)** and their Disease activity index (**c**) were monitored and presented as mean ±SEM, analyzed by two-way ANOVA (with discrete time point and genotype as the independent variables) utilizing Tukey's test to account for multiple comparisons. (**d)** Representative images of WT (left) and *Hsf1* null (right) colons of AOM-DSS treated mice at day 15 (top panels) or day 20 (lower panels). Scale bar - 1 cm. **(e-k)** FACS analysis of the innate immune profile of the EF fraction of colons from WT and *Hsf1* null mice following 15 days (n=5 or 7 for WT or *Hsf1* null (respectively) AOM-DSS treated mice; n=6 for sham treatment (ctrl), for both genotypes, combined from 2 independent experiments) **(e-h)** and 20 days (n=4 or 3 WT or *Hsf1* null (respectively) AOM-DSS treated mice; n=2 or 3 WT or *Hsf1* null (respectively) sham treated (ctrl) mice, from one experiment, representative of two independent experiments). **(i-k)** of the AOM-DSS protocol, or sham treatment (ctrl). The samples were normalized to WT Ctrl. **(e, i)** neutrophils ($CD45^+CD11b^+Ly6G^+$); **(f, j)** macrophages ($CD45^+CD11b^+Ly6G^-F4/80^+$), and **(g,k)** monocytes ($CD45^+CD11b^+F4/80^-Ly6C^+$) gated from the live cell population. Results are presented as mean ± SEM. Two-way ANOVA was performed with Bonferroni correction for multiple comparisons. For **(i)** and **(j)** no significant differences were found. **(h)** Representative FACS plots from day 15-treated colons showing the gating strategy.

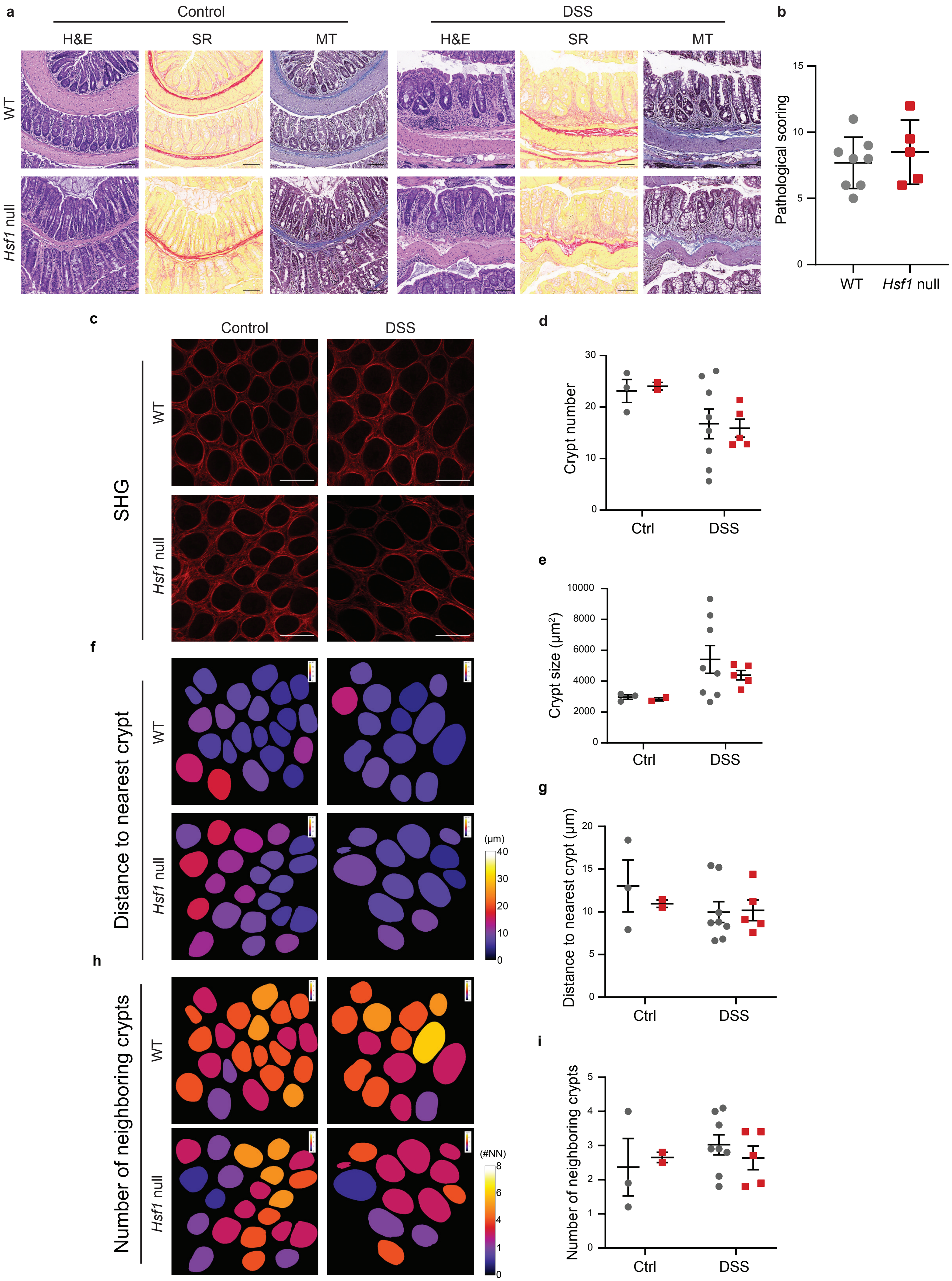
a
Control
DSS
H&E
SR
MT
WT
Hsf1 null
b
Pathological scoring
15
10
5
0
WT
Hsf1 null
c
Control
DSS
SHG
WT
Hsf1 null
d
Crypt number
30
20
10
0
Ctrl
DSS
e
Crypt size (μm²)
10000
8000
6000
4000
2000
0
Ctrl
DSS
f
Distance to nearest crypt
WT
Hsf1 null
(μm)
40
30
20
10
0
g
Distance to nearest crypt (μm)
20
15
10
5
0
Ctrl
DSS
h
Number of neighboring crypts
WT
Hsf1 null
(#NN)
8
6
4
1
0
i
Number of neighboring crypts
5
4
3
2
1
0
Ctrl
DSS

Supplementary Figure 5: HSF1 status does not affect inflammation-mediated changes to the ECM in response to an acute DSS treatment. WT and *Hsf1* null mice treated with 1.5% DSS or water (control) for 7 days were sacrificed 7 days after the end of treatment. n=3 WT mice and n=2 *Hsf1* null mice in water treated groups; n=8 WT and n=5 *Hsf1* null mice in DSS treated groups **(a-b)** Colons were harvested, fixed, and stained with H&E, Sirius red (SR) and Masson's trichrome (MT) staining. **(a)** Representative images from WT (upper panels) and *Hsf1* null (lower panels) mice are shown. Scale bar – 100 µm; **(b)** Colon sections stained with H&E were scored for inflammation by a pathologist (see methods). Results are shown as mean ± SEM analyzed by Student's t-test and found to be non-significant. **(c-i)** Representative cross-sections of the colons with second harmonic generation (SHG) images **(c)** and analysis **(d-i)** of fibrillar collagen of mouse colons following acute DSS treatment or control. Scale bar – 100 µm. **(d-e)** Quantification of the average crypt number **(d)** and size **(e)**. **(f-g)** Analysis of the average distance to the nearest crypt, within 40 µm, calculated based on the SHG images. Representative distance heatmaps of the SHG images presented in **(c)** are shown in **(f). (h-i)** Analysis of the number of neighboring crypts within 20 µm, calculated based on the SHG images. Representative distance heatmaps of the SHG images presented in **(c)** are shown in **(h).** #NN – number of nearest neighbors. Results for **(d-e)**, **(g)** and **(i)** are presented as mean ± SEM, analyzed by two-way ANOVA and found to be non-significant.

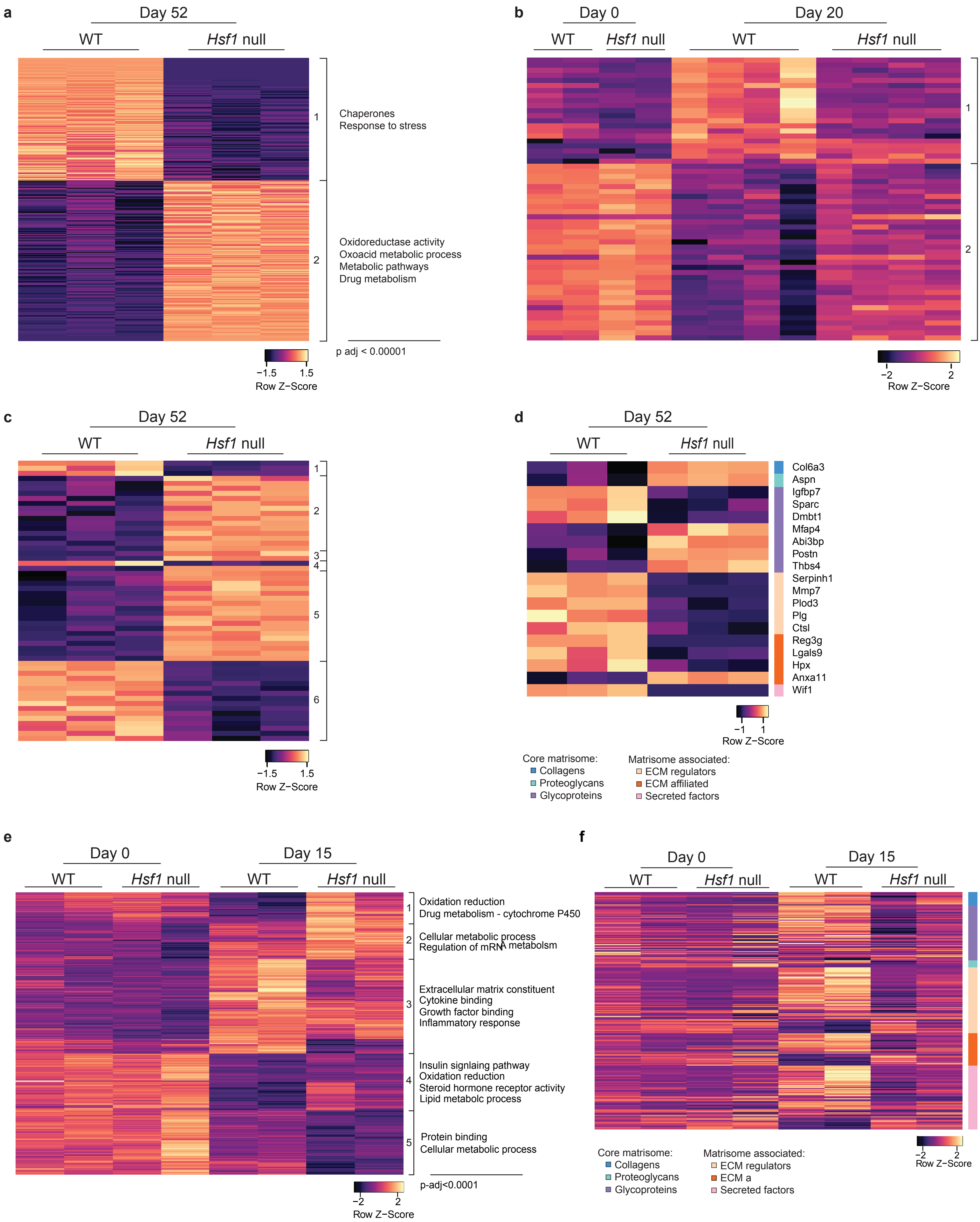

a
Day 52
WT
Hsf1 null
1
Chaperones
Response to stress
2
Oxidoreductase activity
Oxoacid metabolic process
Metabolic pathways
Drug metabolism
p adj < 0.00001
−1.5 1.5
Row Z−Score
b
Day 0
WT
Hsf1 null
Day 20
WT
Hsf1 null
1
2
−2 2
Row Z−Score
c
Day 52
WT
Hsf1 null
1
2
3
4
5
6
−1.5 1.5
Row Z−Score
d
Day 52
WT
Hsf1 null
Col6a3
Aspn
Igfbp7
Sparc
Dmbt1
Mfap4
Abi3bp
Postn
Thbs4
Serpinh1
Mmp7
Plod3
Plg
Ctsl
Reg3g
Lgals9
Hpx
Anxa11
Wif1
−1 1
Row Z−Score
Core matrisome:
Collagens
Proteoglycans
Glycoproteins
Matrisome associated:
ECM regulators
ECM affiliated
Secreted factors
e
Day 0
WT
Hsf1 null
Day 15
WT
Hsf1 null
1
Oxidation reduction
Drug metabolism - cytochrome P450
2
Cellular metabolic process
Regulation of mRNA metabolsm
3
Extracellular matrix constituent
Cytokine binding
Growth factor binding
Inflammatory response
4
Insulin signlaing pathway
Oxidation reduction
Steroid hormone receptor activity
Lipid metabolic process
5
Protein binding
Cellular metabolic process
p-adj<0.0001
−2 2
Row Z−Score
f
Day 0
WT
Hsf1 null
Day 15
WT
Hsf1 null
Core matrisome:
Collagens
Proteoglycans
Glycoproteins
Matrisome associated:
ECM regulators
ECM a
Secreted factors
−2 2
Row Z−Score

Supplementary Figure 6: Similar trends of protein expression are observed at day 20 and day 52 of the AOM-DSS treatment. (a) Heatmap of standardized mass spectrometry values of differentially expressed (DE) proteins (FC > 1.5; FDR<0.1) between Day 52 WT and *Hsf1* null colons, clustered by expression pattern into 2 clusters. Pathway analysis was performed using gProfiler and selected significant pathways are shown. See also Supplementary Data 3-4. **(b)** Heatmap of standardized mass spectrometry values of Day 0-20 DE proteins, which were also DE between Day 52 WT and *Hsf1* null colons, ordered by the Day 52 clusters. **(c)** Heatmap of standardized mass spectrometry values of Day 52 DE proteins, which were also DE at Day 0-20, ordered by Day 0-20 clusters. **(d)** Heatmap of standardized mass spectrometry values of DE matrisome proteins (FC>1.5; FDR<0.1) between Day 52 WT and *Hsf1* null colons, clustered by affiliation to core matrisome and matrisome associated groups. **(e)** Heatmap of standardized RNA sequencing values of DE genes (FC>2; FDR<0.05; base mean >5) between Day 0 and day 15, WT and *Hsf1* null colons, clustered by expression pattern into 5 clusters. Pathway analysis was performed using gProfiler and selected significant pathways are shown. See also Supplementary Data 7. **(f)** Heatmap of standardized RNA sequencing values of DE matrisome genes (FC>2; FDR<0.05;base mean >5) between day 0 and day 15, WT and *Hsf1* null colons, clustered by affiliation to core matrisome and matrisome associated groups.

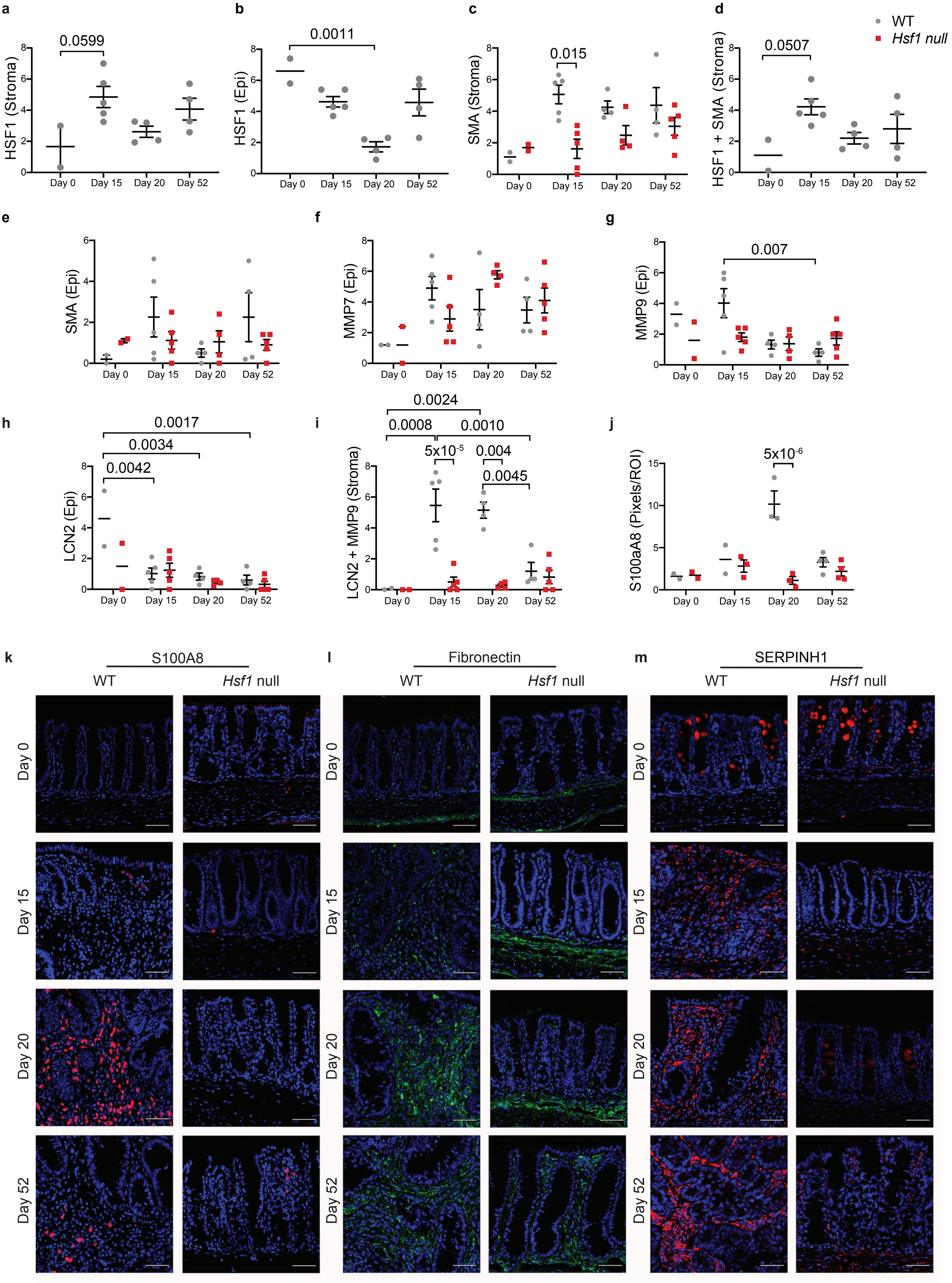

a
b
c
d
WT
Hsf1 null
0.0599
HSF1 (Stroma)
0.0011
HSF1 (Epi)
0.015
SMA (Stroma)
0.0507
HSF1 + SMA (Stroma)
Day 0
Day 15
Day 20
Day 52
e
SMA (Epi)
f
MMP7 (Epi)
g
0.007
MMP9 (Epi)
h
0.0017
0.0034
0.0042
LCN2 (Epi)
i
0.0024
0.0008
0.0010
5x10-5
0.004
0.0045
LCN2 + MMP9 (Stroma)
j
5x10-6
S100aA8 (Pixels/ROI)
k
S100A8
l
Fibronectin
m
SERPINH1
WT
Hsf1 null
Day 0
Day 15
Day 20
Day 52

Supplementary Figure 7: HSF1 expression in SMA-positive stroma correlates with inflammation. Colons from WT and *Hsf1* null mice were excised, fixed, and stained by multiplexed immunofluorescence (MxIF) using antibodies for the depicted proteins, at different time points along the AOM-DSS protocol. n=5 per genotype AOM-DSS treated mice at day 15; n=4 per genotype AOM-DSS treated mice at day 20; n=4 WT and 5 *Hsf1* null mice at day 52; n=2 mice per genotype for control. **(a-i)** The expression of each protein in the stroma or in epithelial cells (epi), and co-expression of the indicated proteins were scored in 5-7 representative images from each mouse, averaged, and are presented for each mouse in the group (see Methods for details). **(j-m)** Colons from WT and *Hsf1* null mice were excised, fixed, and stained for the depicted proteins, at different time points along the AOM-DSS protocol. n=4 mice per genotype at day 52; n=3 mice per genotype at day 20; n=2 WT and 3 *Hsf1* null mice at day 15; n=2 control mice per genotype. **(j-k**) Quantification **(j)** and representative images overlain with DAPI **(k)** of S100A8 staining at the different time points are shown. **(l-m)** Representative images of Fibronectin **(l)** and SERPINH1 **(m)** staining overlaid with DAPI at the different time points are shown. Results are shown as mean ± SEM analyzed by one way Anova **(a, b, d)** and two-way anova and Bonferroni correction for multiple comparisons **(c, e,-j)**. Scale bar – 50 µm.

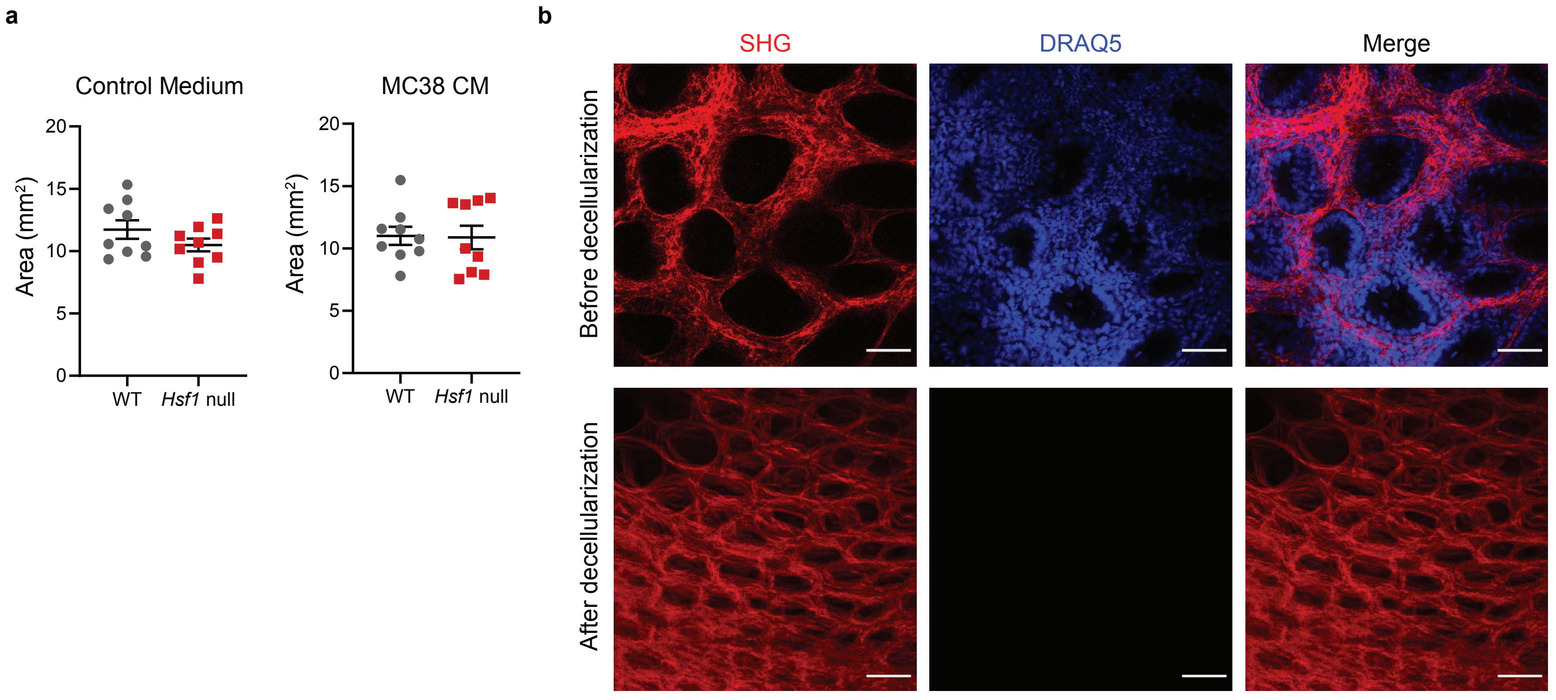

Supplementery Figure 8: Decellularization of colons leads to loss of nuclei. (a) Gel contraction assay was performed in the presence of control medium (RPMI; left panel), or MC38 CM (right panel), and the average area covered by the gel 3 days after seeding was calculated. n=9 mice combined from 3 separate experiments. **(b)** Colon tissues from day 15 AOM-DSS treated mice were imaged by SHG and immunofluorescence (IF) before and after decellularization. Representative SHG + IF images from 3 independent experiments, taken from the mucosal side are shown. SHG is shown in red, DRAQ5 nuclear staining is shown in blue. Scale bar – 50 µm.

	Only DSS			AOM-DSS Day 0		AOM-DSS Day 15			AOM-DSS Day 20			AOM-DSS Day 52		
	**WT**	***Hsf1* null**	**p-val**	**WT**	***Hsf1* null**	**WT**	***Hsf1* null**	**p-val**	**WT**	***Hsf1* null**	**p-val**	**WT**	***Hsf1* null**	**p-val**
**DAI**	0.51 ± 0	0.16 ± 0	ns	0 ± 0	0 ± 0	4.0 ± 3.1	0.7 ± 0.8	ns	1.2 ± 0.6	0.2 ± 0.2	ns	2 ± 0.8	0.4 ± 0.3	ns
**Colonoscopy**	NA	NA	NA	NA		NA			3 ± 2.3	0.5 ± 0.5	0.0204	6.2 ± 2.8	2.1 ± 1.2	0.0054
**Pathology-Inflammation (total)**	7.7 ± 1.9	8.5 ± 2.4	ns	0	0	12.3 ± 2.3	6.6 ± 2.4	0.0006	9 ±1.9	5.1 ± 2.6	0.0071	NA		
**Tissue Involvement (0-4)**	1.2 ± 0.4	1.2 ± 0.4	ns	0	0	2.6 ± 1.1	1.2 ± 0.4	0.0339	1.9 ± 0.8	1 ± 0.6	0.0360	NA		
**Crypt Damage (0-3)**	2.5 ± 0.7	2.5 ± 0.5	ns	0	0	3 ± 0	2.3 ± 0.6	0.0479	2.2 ± 0.5	1.6 ± 1	ns	NA		
**Inflammation (0-3)**	1.3 ± 0.4	1.5 ± 0.5	ns	0	0	2.6 ± 0.6	0.9 ± 0.7	0.0049	2 ± 1.3	0.8 ± 0.8	0.0010	NA		
**Layers (0-3)**	1.2 ± 0.5	1.5 ± 0.5	ns	0	0	1.9 ± 0.4	0.9 ± 0.5	0.0118	1.6 ± 0.5	0.6 ± 0.5	0.0027	NA		
**Regeneration (0-3)**	1.4 ± 0.5	1.8 ± 0.8	ns	0	0	2.4 ± 0.2	1.3 ± 0.4	0.0012	1.2 ± 0.4	1 ± 0.6	ns	NA		
**Pathology-Tumor**														
**Dysplasia**	0	0	ns	0	0	60%	0		20%	0		0	62.5%	
**Adenoma**	0	0	ns	0	0	0	0	0.0384	0	0	ns	78%	37.5%	0.0183
**Carcinoma**	0	0	ns	0	0	0	0		0	0		22%	0	
**Tumor Burden**	NA			NA		NA			NA			127 ± 100	38 ± 23	0.0465

*P values (P val) were determined using two-sided Mann-Whitney test for colonoscopy, two-sided Students' t-test for DAI and tumor burden scores, tissue involvement, crypt damage, inflammation, layers and regeneration, two-way ANOVA for pathology inflammation scores (total), and two-sided Chi squared test for pathology tumor scores.
** NA – not applicable
*** ns – not significant

Supplementary Table 1. Pathological scoring of DSS or AOM-DSS treated mice and non-treated controls

Ab	Conjugation	Clone	Dilution	Source	Cat#	Method	Target species
αSMA	-	1A4	1:1000	Sigma Aldrich	A2547	MxIF	Mouse, Human
HSF1	-	-	1:800	Cell Signaling	4356S	MxIF	Mouse, Human
			1:50			IHC	
LCN2	-	EPR21092	1:800	abcam	ab216462	MxIF	Mouse
MMP7	-	-	1:400	abcam	ab5706	MxIF	Mouse, Human
			1:100			IF	
MMP9	-	-	1:600	abcam	ab38898	MxIF	Mouse, Human
			1:100			IF	
S100A8	-	EPR3554	1:400	abcam	ab92331	MxIF	Mouse, Human
FN1	-	F14	1:400	abcam	ab45688	MxIF	Mouse, Human
			1:100			IF	
SERPIN1	-	EPR4217	1:400	abcam	ab109117	MxIF	Mouse, Human
Goat anti-Rabbit	HRP	polyclonal	1:400	Jackson	111-035-144	MxIF	Rabbit
Goat anti-Mouse	HRP	polyclonal	1:400	abcam	ab97040	MxIF	Mouse
Opal 520 Reagent Pack	Opal 520	-	1:400	Akoya biosciences	FP1487001KT	MxIF	-
Opal 570 Reagent Pack	Opal 570	-	1:400	Akoya biosciences	FP1488001KT	MxIF	-
Opal 620 Reagent Pack	Opal 620	-	1:400	Akoya biosciences	FP1495001KT	MxIF	-
Opal 650 Reagent Pack	Opal 650	-	1:400	Akoya biosciences	FP1496001KT	MxIF	-
Opal 690 Reagent Pack	Opal 690	-	1:400	Akoya biosciences	FP1497001KT	MxIF	-
DAPI	360	-	1:1000	BioLegend	422801	MxIF	-
1X Plus Amp Diluent	-	-	-	Akoya biosciences	FP1498	MxIF	-
CD45	BV-711	30-F11	1:100	BioLegend	103147	FACS	Mouse
F4/80	AF-488	BM8	1:100	BioLegend	123108	FACS	Mouse
CD11b	APC-Cy7	M1/70	1:100	BioLegend	101226	FACS	Mouse
CD11c	APC	N418	1:100	BioLegend	117310	FACS	Mouse
Ly6C	PerCP/Cy5.5	HK1.4	1:100	BioLegend	128011	FACS	Mouse
Ly6G	PE/Dazzle 594	1A8	1:100	BioLegend	127648	FACS	Mouse
Ghost dye	Violet 450	-	1:1000	TONBO	13-0863-T100	FACS	Mouse
CD16/32	-	93	1:100	BioLegend	101320	FACS	Mouse
AF647	AF 647	-	1:200	Jackson	711-605-152	IF	Rabbit
DRAQ5	647	-	1:1000	abcam	Ab108410	SHG	-

Supplementary Table 2. Antibodies and reagents for immunofluorescence and FACS
