# Supplementary material for: Heat Shock Factor 1-dependent extracellular matrix remodeling mediates the transition from chronic intestinal inflammation to colon cancer
Source: Nat Commun. 2020 Dec 7;11:6245. doi: 10.1038/s41467-020-20054-x (PMC7721883; doi:10.1038/s41467-020-20054-x)
Supplement: Supplementary file 3 — Descriptions of Additional Supplementary Files [file 41467_2020_20054_MOESM3_ESM.pdf]

Descriptions of Additional Supplementary Files

Supplementary Data 1

Description: Raw, unfiltered mass spectrometry data. This file contains two sheets - one for day 0-20 and one for day 52.

Supplementary Data 2

Description: List of differentially expressed proteins between WT and Hsf1 null colons presented in Figure 5A.

Supplementary Data 3

Description: Differentially expressed proteins found by the mass-spec analysis. This table contains two sheets, divided by day (0- 20 and 52). Data of day 0-20 is sub-divided by pair-wise (t-test) comparisons, day 52 is subdivided to up/down regulated proteins.

Supplementary Data 4

Description: Pathway analysis of the mass-spec data conducted through the gProfiler database. The first 6 sheets describe DE protein of day 0-20, divided by cluster. The last two describe DE proteins at day 52, divided by expression pattern.

Supplementary Data 5

Description: RNA-sequencing of colons from WT and Hsf1 null mice from Day 0 and Day 15 of the AOM-DSS protocol

Supplementary Data 6

Description: Differentially expressed genes between WT and Hsf1 null mice from Day 0 and Day 15 of the AOM-DSS protocol. This Table contains 3 sheets. Sheet 1 includes all differentially expressed genes with padj=1 and base mean >5; Sheet 2 contains the significantly DE genes between WT and hsf1 null at day 0; and Sheet 3 contains the significantly DE genes between WT and hsf1 null at day 15

Supplementary Data 7

Description: Pathway analysis of the RNA-seq clustered data using the gProfiler webtool. This Table contains 5 sheets, one for each cluster.

Supplementary Data 8

Description: Expression of matrix proteins in the mass-spec data. This table contains 4 sheets, referring to Figures 5C, 5D, S6D and 7C.

Supplementary Data 9

Description: Expression of matrix protein-coding genes in the RNA-seq data. This Table contains 4 sheets. Sheets 1-2 include all the detected (1) and differentially expressed (DE; 2) genes encoding

proteins of the mouse matrisome. Sheets 3-4 include all the detected (3) and DE (4) genes encoding proteins of the mouse colon matrisome.

Supplementary Data 10

Description: Overlap between our list of differentially expressed matrisome proteins and matrisome proteins found to be positively or negatively correlated with disease score (DS) in Pearce et al. (2018). This Table contains two sheets, for positively and negatively correlated proteins, respectively.
